# Modern Beijing sublineage of *Mycobacterium tuberculosis* shift macrophage into a hyperinflammatory status

**DOI:** 10.1080/22221751.2022.2037395

**Published:** 2022-03-01

**Authors:** Jingfeng Tong, Lu Meng, Cheng Bei, Qingyun Liu, Min Wang, TingTing Yang, Howard E. Takiff, Shuye Zhang, Qian Gao, Chuan Wang, Bo Yan

**Affiliations:** a Key Laboratory of Medical Molecular Virology (MOE/NHC/CAMS), School of Basic Medical Sciences, Shanghai Medical College and Shanghai Public Health Clinical Center, Fudan University, Shanghai, People’s Republic of China; b The Center for Microbes, Development and Health, Key Laboratory of Molecular Virology & Immunology, Institut Pasteur of Shanghai, Chinese Academy of Sciences/University of Chinese Academy of Sciences, Shanghai, People’s Republic of China; c Shanghai Public Health Clinical Center, Fudan University, Shanghai, People’s Republic of China; d Department of Tuberculosis Control and Prevention, Shenzhen Nanshan Centre for Chronic Disease Control, Shenzhen, People’s Republic of China; e Instituto Venezolano de Investigaciones Cientificas, Caracas, Venezuela

**Keywords:** Mycobacterium tuberculosis, modern Beijing sublineage, virulence, inflammation, macrophage

## Abstract

The high prevalence of the modern Beijing sublineage of *Mycobacterium tuberculosis* may be related to increased virulence, although the responsible mechanisms remain poorly understood. We previously described enhanced triacylglycerol accumulation in modern Beijing strains. Here we show that modern Beijing strains grow faster *in vitro* and trigger a vigorous immune response and pronounced macrophage infiltration. Transcriptomic analysis of bone marrow derived macrophages infected with modern Beijing lineage strains revealed a significant enrichment of infection, cholesterol homeostasis and amino acid metabolic pathways. The upregulation of proinflammatory / bactericidal cytokines was confirmed by RT–PCR analysis, which is also in consistent with the reduced bacterial burden in modern strains infected macrophages. These results suggest that modern Beijing strains elicit a hyperinflammatory response which might indicate a stronger virulence and contribute to their extensive global prevalence.

## Introduction

*Mycobacterium tuberculosis* (MTB) Lineage 2, known as the Beijing lineage, is one of the most prevalent and widespread lineages worldwide [1]. The Beijing lineage evolved into the ancient and modern sublineages, distinguished by the presence of an IS6110 insertion(s) into the NTF region, but the modern sublineage was more extensively transmitted and became dominant [1,2]. Transmissibility is thought to reflect bacterial virulence, so delineating the factors associated with the high virulence of the modern Beijing sublineage should help understand the basis for its high prevalence.

Although the two sublineages have been shown to have distinct levels of virulence, studying the virulence of MTB lineages in humans is challenging and different studies have often obtained discrepant results [3–7]. In a previous collaborative study, we have confirmed that Beijing family strains are generally poor in cytokine production compared with H37Rv, and found a clear heterogenicity among non-Beijing modern and ancient families [8]. However, in a latter study an overall reduced cytokine level is demonstrated to be associated with modern lineages [9]. Nonetheless, these studies either failed to detect a significant difference of induced cytokine production between modern (Bmyc10 and Bmyc25) and ancient (Bmyc4) Beijing sublineages [8] or have not performed the direct comparation [9]. To better address this issue, an *in vivo* study utilizing mouse pulmonary infection model has demonstrated that modern Beijing sublineage in general are more virulent than the ancient Beijing sublineage, with significantly lower survival cure rates, higher bacterial loads, more inflammatory lesions and increased inflammatory cell infiltration in the lung [5]. Conversely, another *in vivo* study in guinea pigs found that a strain of the ancient Beijing sublineage (RD207) induces more severe inflammation and necrosis [6]. Studies using peripheral blood mononuclear cells or monocyte derived macrophages/dendritic cells have also shown either that the modern Beijing sublineage induces lower levels of pro-inflammatory cytokines or there is no significant difference between the inflammatory responses provoked by the two sublineages [4,7].

Considering the existence of discrepant findings, we decided to select representative strains based on their distinct positions in our previously improved phylogenetic tree of Beijing family to re-investigate the immune responses induced by the two sublineages, and look for patterns suggesting increased virulence that might explain the markedly greater global prevalence of the modern sublineage.

## Materials and methods

### Selection of clinical strains and phylogenetic construction

Three ancient and four modern representative Beijing strains are isolated from patients with pulmonary tuberculosis. They were selected based on their distinct positions in our previously improved phylogenetic tree of Beijing family from the collection of the Shanghai Public Health Clinical Center (SHPHCC) at Fudan University [10]. Genomic DNA extraction and phylogenetic construction were performed as previously described [11].

### Strains and culture

All strains were cultured as previously described [11].

### Cell culture and infection

Bone-marrow derived macrophages (BMDMs) were generated by culturing bone marrow cells (C57BL/6 mice) in complete DMEM medium (10% FBS, 20 ng/ml recombinant mouse M-CSF) for six days. BMDMs were infected with the different strains using multiplicities of infection (MOI) of three for specific period of time according to different assays.

### Cytokine analysis using Luminex assay

Filter sterilized supernatants were assayed by the Cytokine & Chemokine 36-Plex Mouse ProcartaPlex Panel 1A kit (ThermoFisher), according to the manufacturer’s instructions, and analyzed on a Luminex 200 instrument.

### Animals

C57BL/6 mice were purchased from the Department of Laboratory Animals of the Shanghai Institute of Planned Parenthood Research and kept in the specific-pathogen free animal facility of SHPHCC, in compliance with laboratory animal guidelines for ethical review of animal welfare (GB/T 35823-2018). All mice experiments in this study were approved (2021-A011-01) and conducted at SHPHCC.

### Peritoneal immune cell recruitment assay

Mice were intraperitoneal injected with 0.5 ml filtered supernatant. Peritoneal cells were harvested 6 h later, immune stained and analyzed by flow cytometry.

### Flow cytometry

Peritoneal cells were incubated with the fluorescence-conjugated antibodies (Abs) and incubated on ice for 30 min. After washing with PBS twice, the live/dead Abs were added and the cells were incubated on ice for another 20 min. The cells were then washed with PBS and analyzed by flow cytometry. The Mouse Abs used were: CD3-PerCP-Cy5.5 (1:40 dilution, 17A2, Cat.560527, BD), CD4-APC-Cy7-A (1:50 dilution, GK1.5, Cat.561830, BD), B220-Alexa Fluor (1:50 dilution, RA3-6B2, Cat.557957, BD), NK1.1-PE-Cy7-A（1:50 dilution, PK136, Cat.2061509, Invitrogen）, F4/80-APC-A (1:50 dilution, BM8, Cat.2054484, Invitrogen), CD11c (1:50 dilution, N418, Cat.69-0114-80, Invitrogen), CD11b (1:50 dilution, M1/70, Cat.11-0112-82, Invitrogen), Ly6G-PE-A (1:100 dilution, RB6-8C5, Cat.2049427, Invitrogen), and Live / dead-eF455 (1:1000 dilution, Cat.65-08-68-14, Invitrogen). All Abs were purchased from You Ning Wei company. Flow cytometry was performed on a Fortessa instrument (Becton Dickinson, Franklin Lakes, New Jersey, US) and results analyzed using the FlowJo software.

### RNA-Seq sample preparation

Cell pellets were re-suspended in 1 mL TRIzol reagent and subjected to bead-beating (0.1 mm silica beads) for 30 s, 3 times. The samples were then centrifuged to sediment the beads and cell debris and the supernatants were transferred to phase lock gel tubes, to which chloroform (400 μL) was added. The tubes were mixed by vigorous inversions, centrifuged, and the upper, acqueous phase was transferred to a new tube containing 450 μL isopropanol. The precipitated RNA was pelleted by centrifugation, washed with 80% ethanol followed by pure ethanol and re-suspended in 50 μL of RNAase free water. The quality and integrity of the total RNA was checked by NanoDrop 2000 and electrophoresis. cDNA libraries were constructed for each sample and sequenced on an Illumina HiSeq 2500 sequencer.

### Transcriptome analysis

Sequencing reads were aligned to the GRCm38 mouse reference genome using HISAT2. Unique reads were selected and sorted using SAMtools, then quantitated using htseq-count. After filtering genes with low expression, the counts were normalized using DESeq2. The ComBat method from the sva package in R was used to remove the batch effect [12]. Correlations between samples were determined by Pearson’s correlation coefficient of the logarithm of the counts. Principal Component Analysis (PCA) was performed with the scatterplot3d package in R. Differentially Expressed Genes (DEGs) in mouse macrophages infected with modern compared with ancient Beijing strains were identified using the limma package (v3.44.3) with a cutoff *p* value < 0.05, and | fold change | > 1.5. The host responses to infection with the respective strains were analyzed and compared to the negative control using Gene Set Enrichment Analysis (GSEA) (v4.1.0), based on mouse functions and pathways available in the Gene Ontology (GO) and Kyoto Encyclopedia of Genes and Genomes (KEGG) databases integrated in DAVID (v6.8) (https://david.ncifcrf.gov). Functions and pathways with a *p* value < 0.05 and | NES (normalized enrichment score) | > 1 were considered to be significantly enriched. The data was visualized with Draw Venn Diagram (via http://bioinformatics.psb.ugent.be/webtools/Venn), ggplot2 and Pheatmap in R (v4.0.2). Raw sequencing data was available under accession number PRJNA723409 on NCBI.

### RNA extraction and quantitative reverse-transcription PCR (qRT-PCR)

Total RNA was extracted by TRIzol, and cDNA was synthesized using PrimeScript™ RT reagent Kit (TAKARA) according to the manufacturer’s instructions. qRT-PCR was carried out by CFX96^TM^ Real-Time PCR System (Bio-Rad) with iQ SYBR green supermixture kit (TAKARA). The primer sequences of genes measured were listed in supplementary Table 4.

### CFU assay in BMDMs

BMDMs were plated in 24-well plates at 1×10^5^ cells/well 1 d before infection and then infected with single-cell suspension of 7 clinical strains (4 modern strains and 3 ancient strains) and H37Rv at a MOI of 3. The infected BMDMs were incubated at 37 °C and 5% CO_2_ for 3 days. BMDMs were washed with PBS for 3 times and then lysed with 0.1% Triton X-100 to collect the intracellular MTB bacteria. A serial dilutions of cell lysates were plated on 7H10 + OADC plates, and the plates were incubated at 37°C for 3 weeks before CFU counting.

## Results

### Modern Beijing strains show enhanced fitness *in vitro*

**Figure 1.**
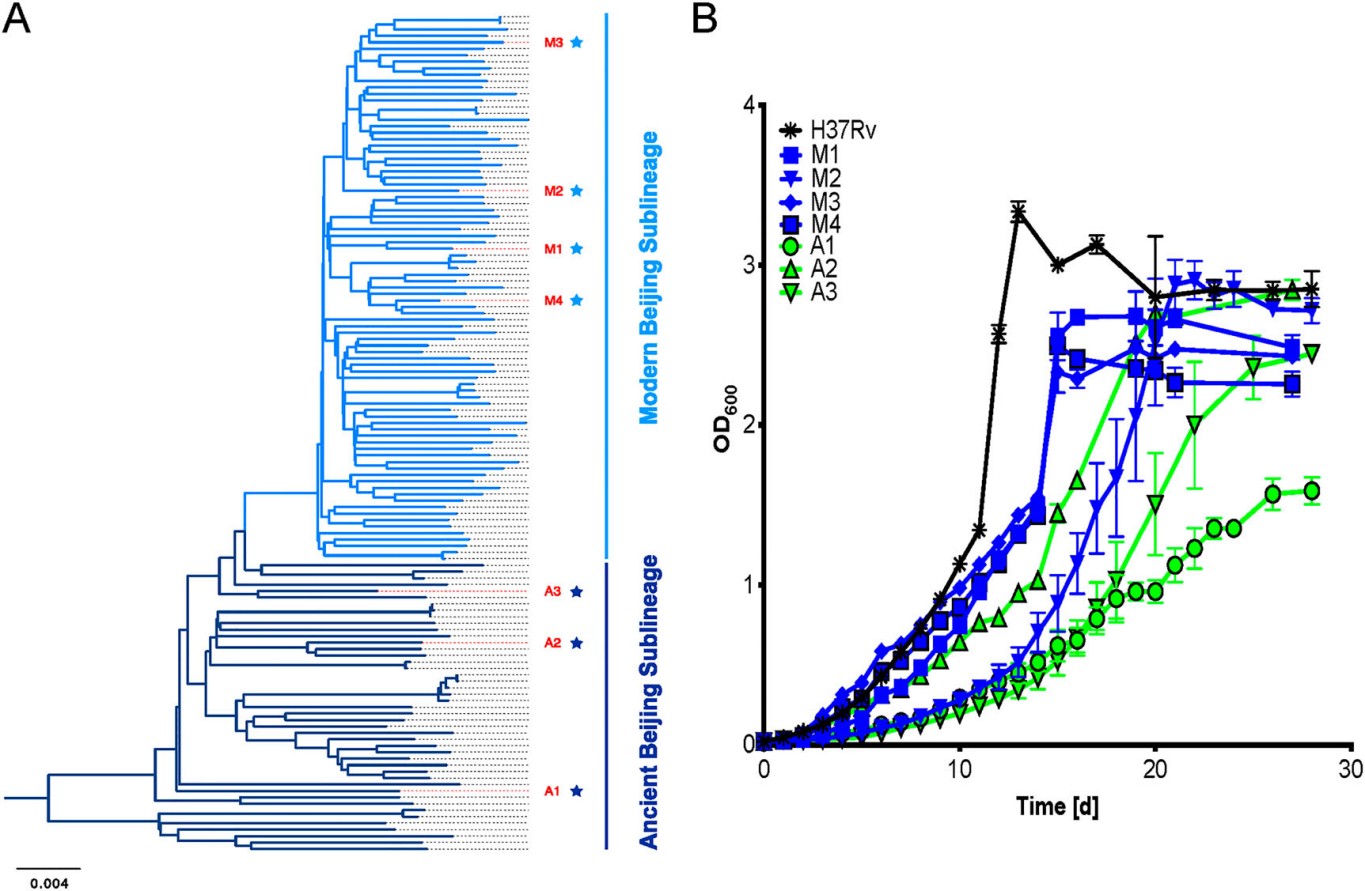
Modern Beijing strains show enhanced fitness in vitro. A, The phylogenetic tree of Beijing family strains of MTB. Dark blue stars represent the three ancient Beijing strains (A1-A3), and light blue stars represent the four modern Beijing strains (M1-M4) used in the study. B, Modern Beijing strains generally grow faster than ancient Beijing strains. To compare growth curves of modern Beijing strains and ancient Beijing strains, the seven representative strains and the laboratory control strain H37Rv were grown in 7H9 broth in vitro and the optical density was measured at the indicated time points.

Four modern and three ancient representative strains, termed M1-4 and A1-3 respectively were selected based on their distinct positions in our previously improved phylogenetic tree of Beijing family [10]. The selected strains were grown in 7H9 media, with H37Rv as a control (Figure 1A). H37Rv showed the fastest growth and entered the stationary phase first, followed by three of the modern strains, with the three ancient strains growing slower than all but the M2 strain (Figure 1B). These results suggested that modern Beijing strains bears a stronger adaptability than ancient Beijing strains *in vitro*.

### Modern Beijing strains induce excessive inflammatory cytokine production in macrophages

**Figure 2.**
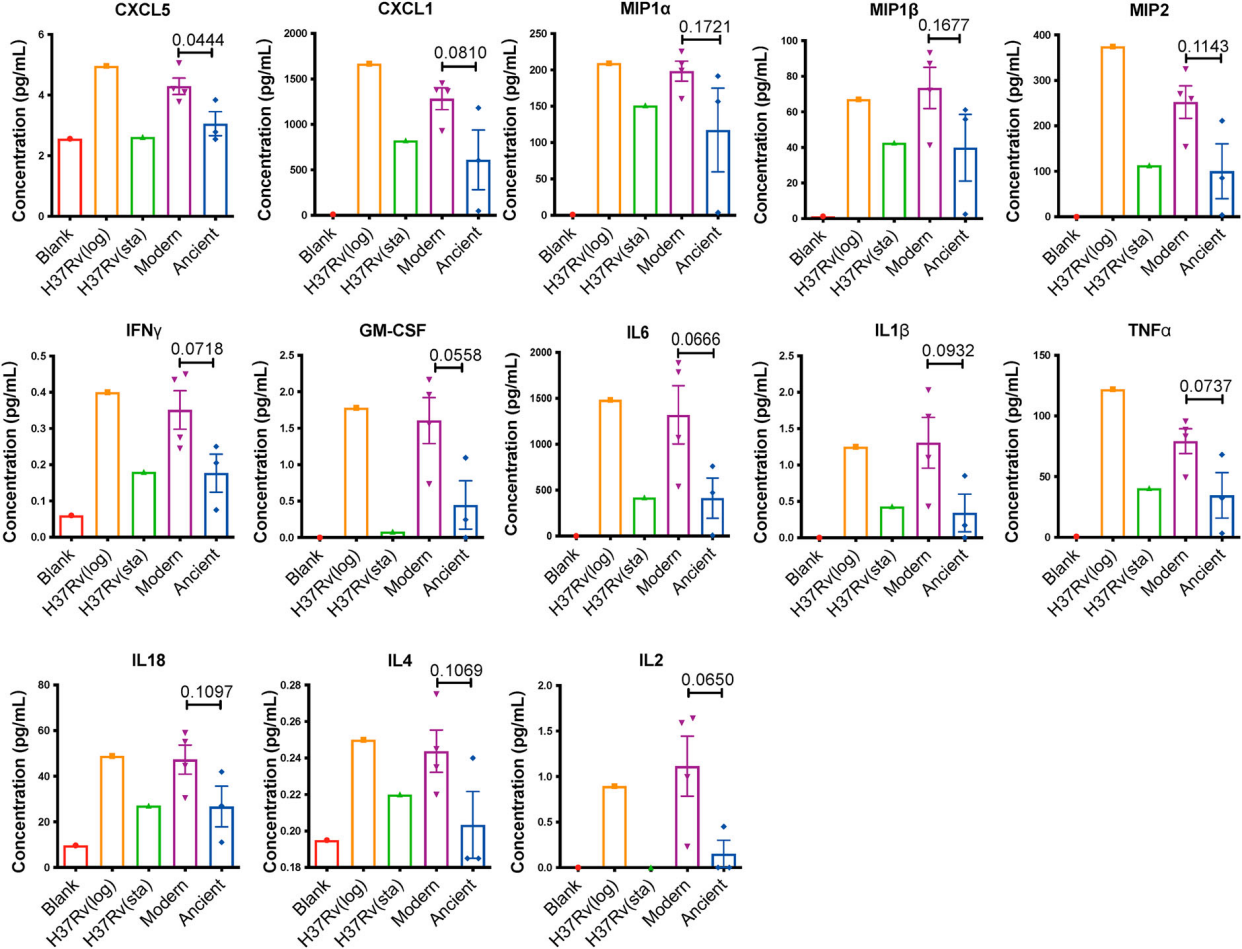
Modern Beijing strains induce greater cytokine responses. BMDMs were infected with log-phase and stational-phase H37Rv or log-phase modern and ancient Beijing strains for 24 h. Cell culture supernatants were collected and analyzed for cytokine/chemokine concentration using Cytokine & Chemokine 36-Plex Mouse ProcartaPlex Panel 1A kit, to detect: CXCL5, CXCL1, MIP1a, MIP1β, MIP2, IFNγ, GM-CSF, IL6, IL1β, TNFa, IL18, IL4, IL2. Data shown are means ± SEM. Unpaired Student’s T test.

Macrophages are the first line of immune defence against MTB infection. To investigate the host immune responses induced by modern and ancient Beijing strains at the very early stage of infection, we profiled the secreted cytokines and chemokines in BMDMs infected with the different strains at 24 hpi. To make sure the bacteria chosen for infection assay have the strongest capability to elicit cytokine and chemokine productions, we first compared and found that the levels of secreted cytokines and chemokines were higher in BMDMs infected with H37Rv from log-phase cultures than with H37Rv from stationary-phase cultures (Figure 2 and supplementary Table 1). Therefore, log-phase cultures were used for all subsequent infections. Strains of the modern Beijing sublineage induced higher levels of pro-inflammatory / bactericidal cytokines and chemokines than the ancient Beijing strains, including CXCL5, CXCL1, IFNγ, GM-CSF, IL6 and IL1β (Figure 2). These results suggested there may be differences in intracellular bacterial survival and the immune cell recruitment elicited by the infections of two sublineages.

### Modern Beijing strains infection cause a reduced intracellular bacterial burden but more extensive macrophage infiltration

**Figure 3.**
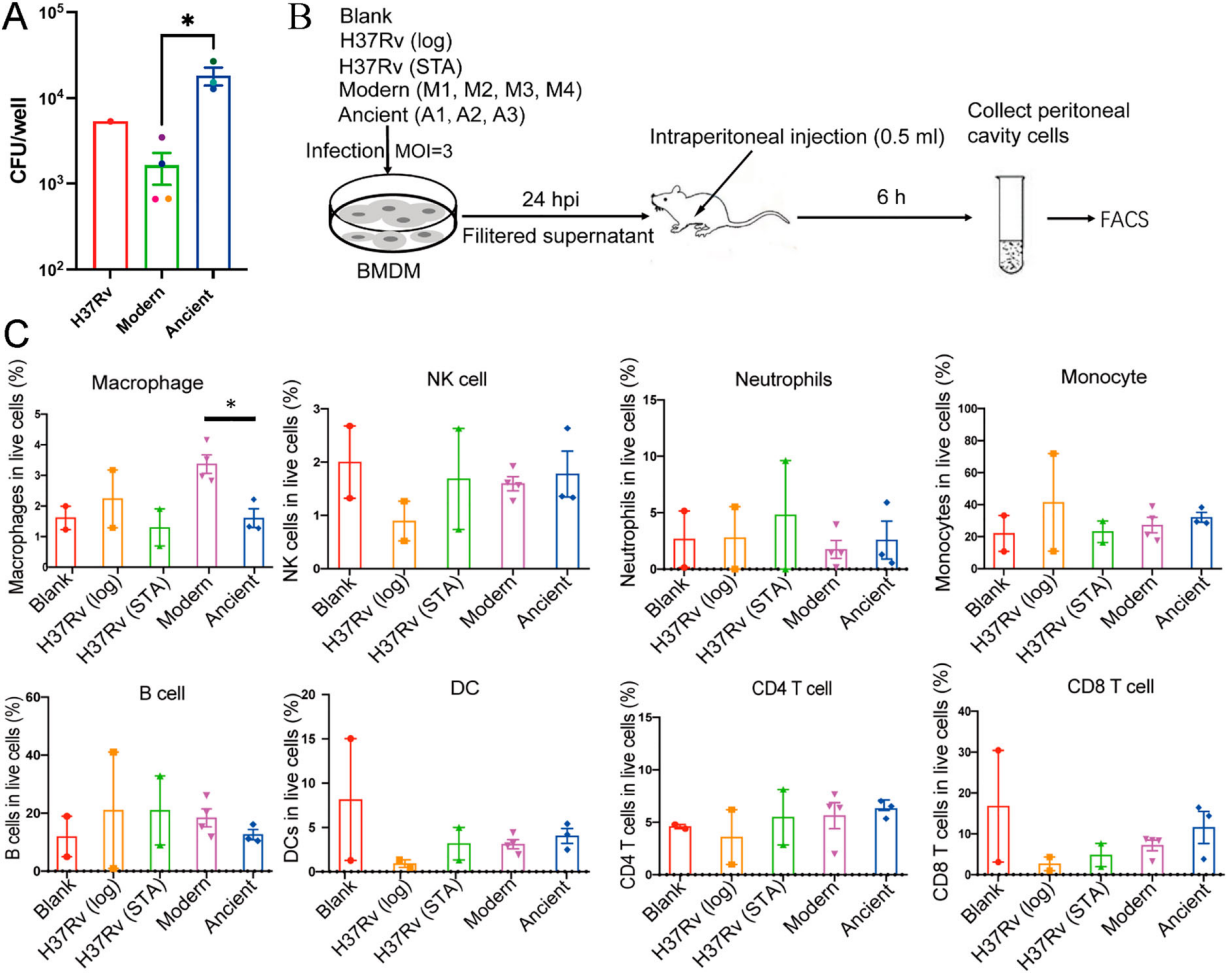
Supernatant from Modern Beijing strains infected BMDMs induce augmented macrophages infiltration in the abdominal cavity. A, CFUs at 3 dpi of H37Rv, modern and ancient Beijing strains infected BMDMs. Data show the mean ± SEM, and each colour represent one strain. B, Flowchart of peritoneal immune cell recruitment assay. BMDMs were uninfected or infected with H37Rv (log) / H37Rv (STA) / modern Beijing strains (M1, M2, M3, M4) / ancient Beijing strains (A1, A2, A3), MOI = 3. Supernatants were collected and filtered at 24 hpi, and 500 µl of each was applied for mice intraperitoneal injection. Then the peritoneal cells were collected at 6 hpi and subsequently stained for flowcytometry. C, The proportion of each immune cells. The values are pooled from two independent experiments (mean ± SEM). * *p* < 0.05, unpaired Student’s T test.

The different intracellular survival capabilities of modern and ancient strains were determined by a macrophage infection assay. It revealed a significantly decreased intracellular bacterial load in modern strains infected macrophages (Figure 3A), which is consistent with the elevated bactericidal cytokines described in Figure 2. To explore differences in cell recruitment, culture supernatants were injected intraperitoneally into C57BL/6 mice, and then after 6 h the peritoneal cells were collected by lavage and subsequently analyzed by flow cytometry (Figure 3B). As expected, the supernatants from modern Beijing strains elicited a significantly higher percentage of macrophages in the total live cells compared to the those from the ancient sublineage strains (Figure 3C and supplementary Figure 1). No differences were observed for CD4 T cells, nature killer cells (NK), neutrophils, monocytes, B cells or dendritic cells (DC). Collectively, these results indicated that the modern Beijing strains induce stronger inflammatory responses with increased macrophage infiltration and reduced intracellular bacterial burden.

### RNA-seq revealed higher infection, cholesterol homeostasis and amino acid metabolism in BMDMs infected with modern Beijing strains

**Figure 4.**
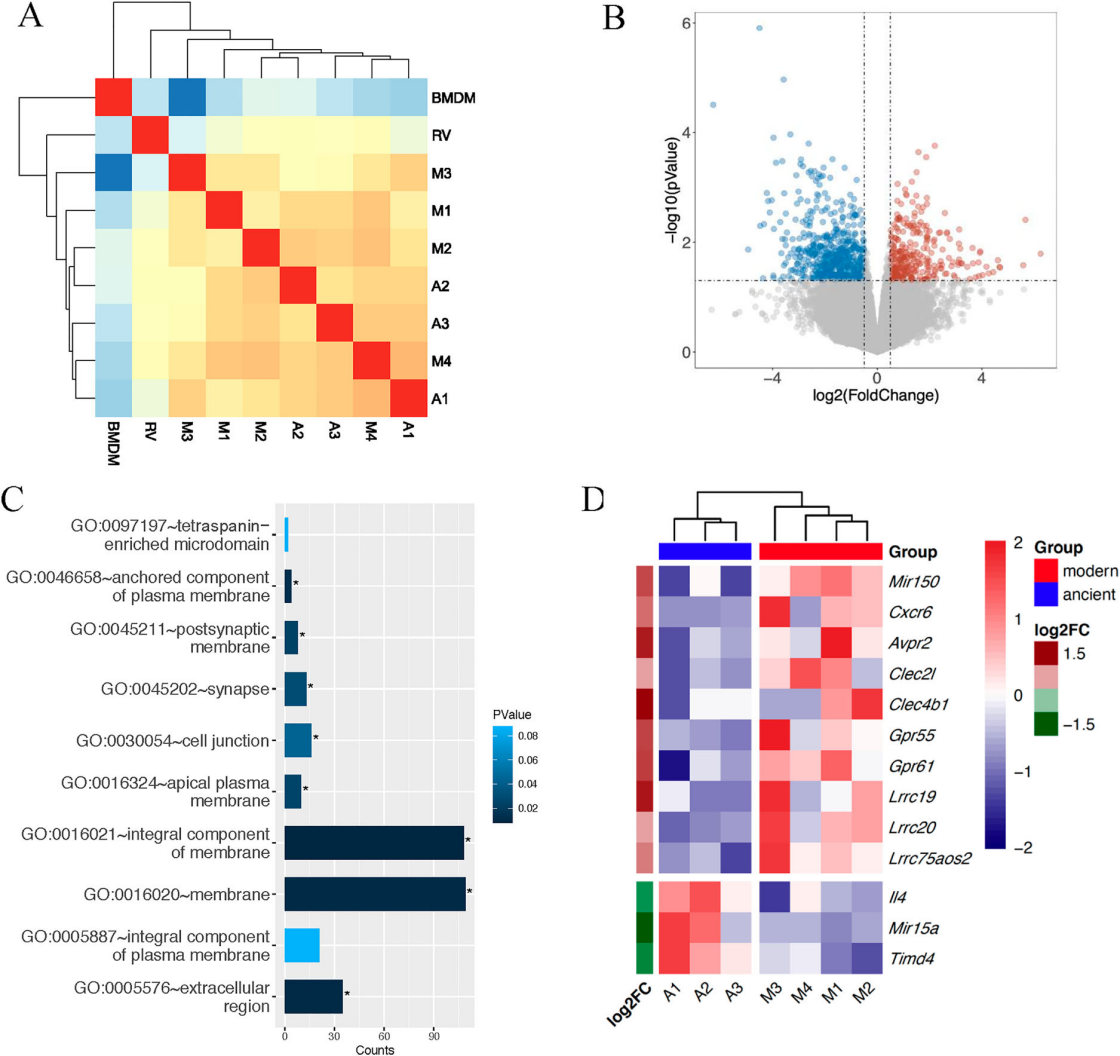
Differential expression analysis. A, Heatmap showing the correlation between pairwise samples. A1-A3 and M1-M4 represent BMDMs infected with modern (M1–M4) and ancient (A1–A3) Beijing strains. RV indicates infection with H37Rv and BMDM indicates the uninfected negative control. B, Volcano plot of mouse BMDM DEGs. Coloured plots stood for genes with significant differences (*p* < 0.05). Red plots stood for up-regulated genes of BMDMs infected by modern strains compared to ancient strains while blue plots stood for down-regulated genes. The x-axis represented log2 of fold change and the y-axis represented the log10 of *p* values. C, GO analysis of cellular component enriched in DEGs. D, Heatmap showing the expression profile of selected DEGs.

We used RNA-seq to study the transcriptional responses in infected mouse BMDMs. The transcriptome profiles clearly separated BMDMs infected with the Beijing strains from uninfected BMDMs or BMDMs infected with H37Rv, but could not distinguish between infections with the two Beijing sublineages (Figure 4A and supplementary Figure 2A). However, differential expression analysis showed that, compared to BMDMs infected with the ancient strains, BMDMs infected with modern strains had 803 DEGs, including 284 up-regulated and 519 down-regulated genes (Figure 4B and supplementary Figure 2B). GO analysis showed that the products of the DEGs were located mainly on the cell membrane and extracellular regions, suggesting that the modern strains induced changes in macrophage secretion and intercellular interactions (Figure 4C).

**Figure 5.**
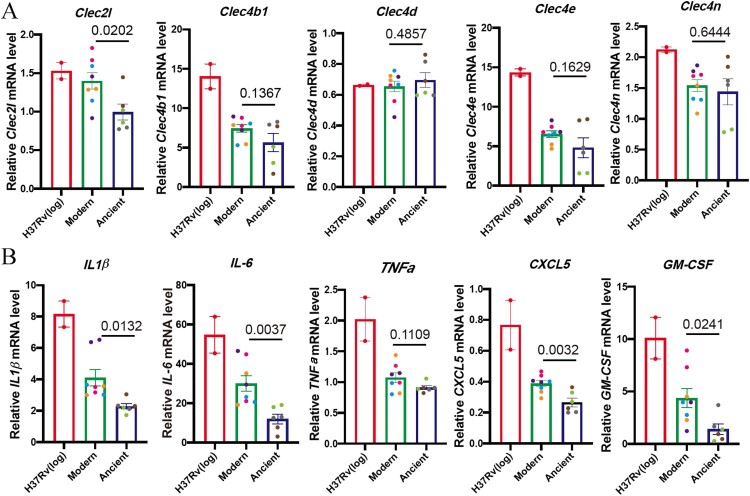
Modern Beijing strains induce elevated CLRs and proinflammatory cytokines and chemokines mRNA expression. BMDMs were infected with log-phase H37Rv, modern and ancient Beijing strains for 24 h. Infected BMDMs were collected and analyzed for the mRNA expression levels for target genes. A, The mRNA expression levels for *Clec2 l, Clec4b1, Clec4d*, *Clec4e* and *Clec4n*. B, The mRNA expression levels for *IL-1β*, *IL-6*, *TNFα*, *CXCL5* and *GM-CSF*.

Several DEGs were consistent with the increased cytokine response described above (Figure 2), including two C-type lectin receptors (CLRs), *Clec4b1* and *Clec2 l*, which were up-regulated in BMDM infected with modern strains (Figure 4D). Clec4b1, along with three other CLRs – Clec4e, Clec4d and Clec4n – were shown to be receptors for mycobacteria [13]. CLRs have been shown to recognize MTB cell envelope glycolipids and stimulate cellular activation, principally via NF-κB, resulting in increased expression of TNFα, IFNγ, IL-6, IL-1β, etc. [13]. The elevated expressions of CLRs and the downstream proinflammatory cytokines and chemokines were also confirmed by qRT-PCR analysis (Figure 5A and B). Other inflammatory response-related genes were also either upregulated or downregulated, including *Lrrc19* and *Timd4*, which positively and negatively, respectively regulate proinflammatory cytokines [14,15].

**Figure 6.**
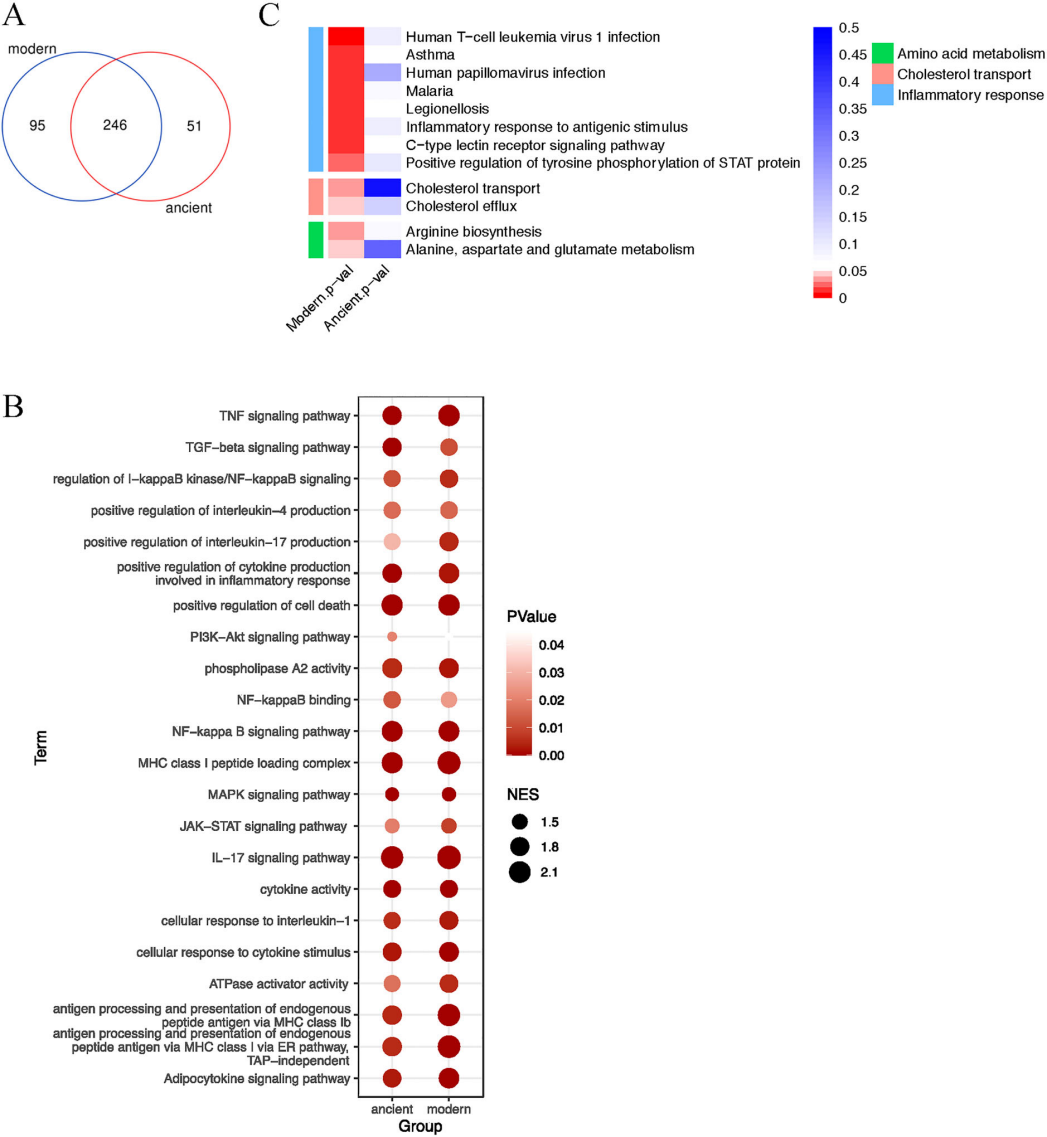
Gene set enrichment analysis. A, Venn diagram showing the number of functions and pathways enriched in BMDMs infected with strains of the modern and ancient Beijing sublineages, analyzed by GSEA. B, Heatmaps of selected enriched functions and pathways shared by modern and ancient strains infected BMDMs. C, Heatmaps of selected unique functions and pathways of BMDMs infected by modern strains.

We then performed GSEA to discover the functions and pathways differentially stimulated by BMDMs infected with the modern Beijing strains (Figure 6A). As expected, GSEA revealed immune responses previously associated with infections, including the IL-17 and NF-κB signalling pathways (Figure 6B). Intriguingly, there was also increased transcription of genes associated with enriched cholesterol transport and cholesterol efflux (Figure 6C). Considering cholesterol as an essential carbon source for MTB, the increased cholesterol metabolism might also contribute to the impair intramacrophage growth and survival of the modern Beijing strains in Figure 3(A). We also found that some amino acid metabolic pathways were differentially regulated between macrophages infected with modern and ancient strains (Figure 6C), consistent with previous reports that host amino acid metabolism is closely linked to infection and inflammation [16]. Taken together, the cytokines / chemokines patterns and RNA-seq results suggest that infection with modern Beijing strains induce greater inflammation and increased cholesterol and amino acid metabolism, which might be the underlying mechanism for increased immune cell infiltration and reduced intracellular bacterial burden.

## Discussion

Modern Beijing strains of MTB are amongst the most prevalent strains worldwide and cause the majority of epidemic outbreaks [1,2], but the determinants responsible for their apparent advantage are largely unknown. Based on our previously improved phylogenetic tree of Beijing family [10], we selected representative strains with distinct phylogenetic position and found that modern Beijing strains grow faster than ancient strains *in vitro*, consistent with their increased accumulation of triacylglycerols that may provide a readily available carbon source [11]. A hyperinflammatory response was uncovered to be associated with modern Beijing strains, which suggesting a stronger virulence compared with ancient strains. The hyperinflammatory response induced by modern Beijing strains might be one of the key factors contributing to their diminished intracellular bacterial burden and help to explain their extensive global prevalence.

To study the impact of infections with modern and ancient strains on macrophages, the primary innate immune cell infected in tuberculosis, we performed cytokine profiling of supernatants from infected mouse BMDMs and found that modern Beijing strains induce greater inflammatory responses. This is consistent with *in vivo* studies in mice, but conflicts with the results from infections of guinea pigs, human PBMCs and monocyte derived macrophages/dendritic cells [4–7]. These discrepancies could be attributed to the selection of different strains, which may also be one of the important facts for discrepant results regarding the innate immune response triggered by other non-Beijing modern and ancient lineages [8,9]. Another variable parameter need to be noted is the application of different animal models or host cells in these studies. Different host genetic background may have a great impact on the dynamic of immune response even to the same strain. Thus, it would be informative to monitor the immune response of different strains in the above studies using the same *in vivo* or *ex vivo* MTB infection model. Last but not the least, the inconsistent findings could just simply due to the different dose / multiplicity of infection, which has been well documented to induce diverse host responses [17,18].

Here we demonstrated that modern Beijing strains grow faster than ancient strains *in vitro*, but a compromised survival capability of modern strains in macrophage was also observed. This might be explained by the distinct environmental stresses between *in vitro* liquid culture condition and macrophage intracellular microenvironment. Normally, 7H9 (with OADC) liquid medium is a nutrient rich environment favouring MTB growth. The increased *in vitro* grow rate of modern Beijing strains is consistent with our previous *in vitro* findings that an increased accumulation of triacylglycerols in modern strains may provide a readily available carbon source [11]. Although MTB can survival within macrophages, host cells preserve multiple strategies to supress the MTB intracellular growth, such as bactericidal cytokines and ROS / NOS signals. Thus, it is easy to understand the reduced intracellular bacterial burdens in modern strains infected macrophages considering the elevated bactericidal cytokines in modern strains infected macrophage. What’s more, the increased cholesterol metabolism in modern Beijingstrains infected macrophages might also impair the intramacrophage growth of modern strains, considering cholesterol as an essential carbon source for MTB. Together, it re-emphasizes the importance to take the experimental settings into account when interpreting the fitness or the virulence of different MTB strains.

The immune cells recruited to the site of infection will organize into a granuloma, the hallmark of tuberculosis, and necrosis of the granuloma and subsequent cavity formation likely promote bacterial transmission. We found that culture filtrates from modern strains elicit greater macrophage infiltration after intraperitoneal injection and BMDM infected with modern strains have higher levels of secreted cytokines / chemokines (Figure 2, Figure 3B-C and Figure 5). The increased cytokines / chemokines will promote the immune cell infiltration into the infection sites, which is closely correlated with stronger virulence and might promote transmission. Similarly, chemokine receptor *Cxcr6* was also up-regulated in BMDM infected with modern Beijing strains (Figure 4D). CXCR6 has been implicated in macrophage and T cell migration, and the deficiency of *Cxcr6* can promote host defence against MTB infection [19]. The increased macrophages and other immune cells infiltration at the very early stage could be harbingers of the accelerated granuloma maturation, which help restrict MTB infection locally and constrain bacterial proliferation. Although the overall immunopathology may be lower induced by modern Beijing strains, the transmission mainly dependents on the progressed granuloma (cavitation) formation. Our finding is consistent with another excellent murine study, in which Verma *et. al.* found the high-transmission strains induced a lower bacterial burden and less cavitation formation [20].

Transcriptome and qRT-PCR analysis showed that modern Beijing strains elicited a significant enrichment of pathways associated with infection, cholesterol homeostasis and amino acid metabolism. But *in vitro* or *in vivo* infection experiment measuring the bacterial burden when the specific pathway is blocked are required to further verify the exact role of those candidate genes or pathways. Interestingly, we found some DEGs, such as the upregulated *Avpr2,* that are not known to be associated with infections, and nearly half (403/803) of DEGs were unidentified predicted genes or pseudogenes (supplementary Figure 2C-D, supplementary Table 2). In addition, several microRNAs were differentially expressed in modern Beijing infected cells (supplementary Table 3). These DEGs could be potentially involved in the host immune regulation during MTB infection.

In summary, our results provide evidences that modern Beijing strains elicit a hyperinflammatory response at the very early stage of infection that may help explain their higher global prevalence. Considering the discrepant results from various studies, we should be caution and always think about the heterogeneity within modern and ancient Beijing strains when interpreting their virulence. Further *in vivo* experiment comparing the same strains in different studies in the same experimental setting are also required to verify our findings. This might be accomplished with competition experiments in which animals are infected with a mixture of modern and ancient strains to see if one strain eventually becomes dominant. These experiments, together with the results described here, should deepen our understanding of the pathological mechanisms of different Beijing strains and identify novel virulence determinants.

## Supplementary Material

Supplemental Material

Supplemental Material

Supplemental Material
