# Supplementary material for: Modern Beijing sublineage of Mycobacterium tuberculosis shift macrophage into a hyperinflammatory status
Source: Emerg Microbes Infect. 2022 Mar 1;11(1):715–24. doi: 10.1080/22221751.2022.2037395 (PMC8890550; doi:10.1080/22221751.2022.2037395)
Supplement: Supplemental Material [file TEMI_A_2037395_SM0015.docx]

**Supplementary Data**


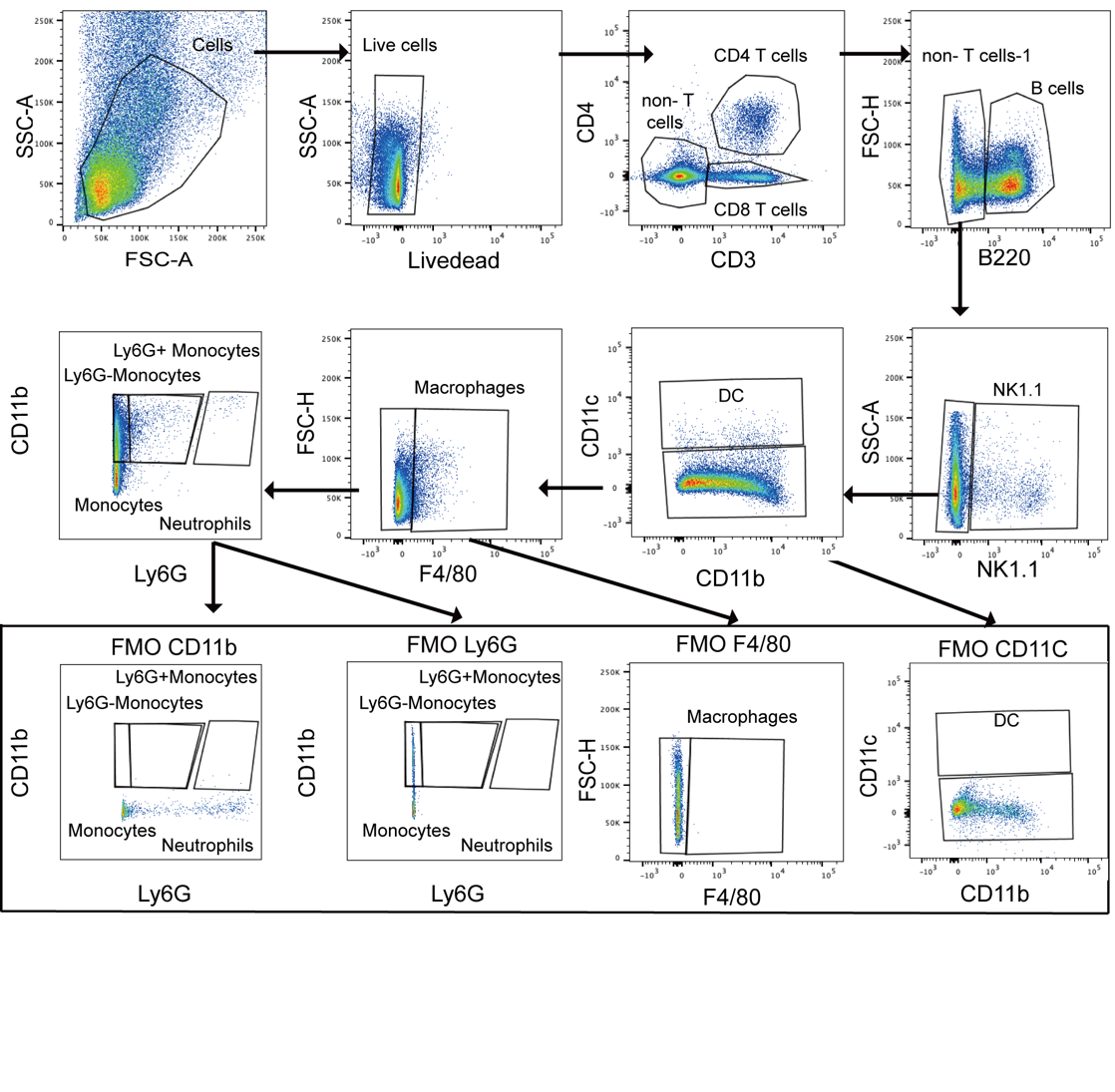


**Figure S1. Gating strategy.**

The peritoneal cells were stained with CD3, CD4, B220, F4/80, NK1.1, CD11b, CD11c and Ly6G and the live populations were gated based on the following strategy: CD4 T cell: CD3+CD4+; CD8 T cell: CD3+CD4-; B cell: CD3-CD4-B220+; NK cell: CD3-CD4-B220-NK1.1+; DC: CD3-CD4-B220-NK1.1-CD11c+; Macrophage: CD3-CD4-B220-NK-CD11c-F4/80+; Monocytes: CD3-CD4-B220-NK1.1-CD11c-F4/80-LY6G^-^&^low^ CD11b+; Neutrophils: CD3-CD4-B220-NK1.1-CD11c-F4/80-LY6G^hig^CD11b+. Fluorescence minus one (FMO) controls were used to gate the respective populations.

**Figure S2. RNA-seq analysis results.**

A, PCA plot of modern strains infection (n=4), ancient strains infection (n=3), H37Rv infection (n=1) and negative control (n=1). B, Heatmap showing the expression profile of DEGs. C, Volcano plot of pseudogenes in DEGs. Colored plots stood for pseudogenes with significant differences (p < 0.05, | FC | > 1.5). D, Heatmap showing the 20 up-regulated and down-regulated pseudogenes with greatest fold changes.

**Table S1. Cytokine/chemokine profile of supernatants from uninfected BMDMs and BMDMs infected with the different strains.**

Refer to attached Table S1 file.

**Table S2. Pseudogenes identified as DEGs.**

Refer to attached Table S2 file.

| **Micro RNA** | **logFC** | **P.Value** | **Function** |
| --- | --- | --- | --- |
| Mir150 | 1.261483679 | 0.016582622 | Suppresses the TLR2 response in macrophages [1]; ﻿ |
|  |  |  | Regulates macrophage apoptosis by targeting the programmed cell death protein-4 (PDCD4) [2]. |
| Mir15a | -1.953715901 | 0.017601246 | ﻿Targets IKKα mRNA and regulates the activation of macrophages via ﻿NF-κB signaling [3]. |
| Mir181a-2 | -1.444927513 | 0.043408885 | A key molecule controlling macrophage polarization and regulating the inflammation response in macrophages. [4-7]; |
|  |  |  | Mir181 family modulates the inflammatory response under variant conditions [8-13]. |
| Mir181c | -2.000846706 | 0.01832866 |  |
| Mir181d | -2.140248016 | 0.037464337 |  |
| Mir466j | -3.050732715 | 0.024947019 | MiR-466j was significantly enriched in some synaptic transmission-related biological processes [14]. |
| Mir6353 | -2.614235359 | 0.01572265 | Unknown |
| Mir6361 | -2.80028325 | 0.04147023 |  |
| Mir6392 | -4.503614056 | 1.24E-06 |  |
| Mir6399 | -2.121574568 | 0.049038201 |  |
| Mir6920 | -0.831307695 | 0.047001106 |  |
| Mir7009 | -1.493428288 | 0.006562631 |  |
| Mir7030 | -2.052407233 | 0.020470797 |  |
| Mir7049 | 5.580044347 | 0.026294035 |  |
| Mir7054 | -1.983542704 | 0.029967237 |  |
| Mir7656 | -2.254212695 | 0.013522946 |  |
| Mir7675 | -1.925911936 | 0.007251609 |  |

**Table S3. MicroRNA identified as DEGs.**

| Primer name | Sequence (5’ – 3’) |
| --- | --- |
| Clec2l RT-F | ATTTGTGCCTCTCAGCTTTCA |
| Clec2l RT-R | TTCCTTGGAGAATTTTACCCACTT |
| Clec4b1 RT-F | ATGTGTCGTAACATATCGCAGC |
| Clec4b1 RT-R | GACTGAACCTGATGCCTCACT |
| Clec4e RT-F | CAGTGGCAATGGGTGGATGA |
| Clec4e RT-R | GTCCCTTATGGTGGCACAGT |
| Clec4d RT-F | CTGGAGCTACAGGAGGTACTTG |
| Clec4d RT-R | TGCTTCGGTGTTGATGGTCA |
| Clec4n RT-F | ACCCAGCAGCTGAATGAGTC |
| Clec4n RT-R | CCAGCCCCATTTCGAAGGAT |
| GM-CSF RT-F | TAAGGTCCTGAGGAGGATGTGG |
| GM-CSF RT-R | GACTTCTACCTCTTCATTCAACGTG |
| TNF𝛼 RT-F | CTGTAGCCCACGTCGTAGC |
| TNF𝛼 RT-R | TTGAGATCCATGCCGTTG |
| IL-6 RT-F | TCTAATTCATATCTTCAACCAAGAGG |
| IL-6 RT-R | TGGTCCTTAGCCACTCCTTC |
| IL-1𝛽 RT-F | TTGACGGACCCCAAAAGA |
| IL-1𝛽 RT-R | GATGTGCTGCTGCGAGATT |
| CXCL5 RT-F | GCCCTACGGTGGAAGTCATA |
| CXCL5 RT-R | GTGCATTCCGCTTAGCTTTC |
| GAPDH RT-F | TGCTGTCCCTGTATGCCTCTG |
| GAPDH RT-R | TTGATGTCACGCACGATTTCC |

**Table S4. qRT-PCR primers.**

**Supplementary Reference**

1. Ghorpade DS, Holla S, Kaveri SV, et al. Sonic hedgehog-dependent induction of microRNA 31 and microRNA 150 regulates Mycobacterium bovis BCG-driven toll-like receptor 2 signaling. Mol Cell Biol. 2013 Feb;33(3):543-56.

2. Wang Z, Kong LC, Jia BY, et al. Analysis of the microRNA Expression Profile of Bovine Monocyte-derived Macrophages Infected with Mycobacterium avium subsp. Paratuberculosis Reveals that miR-150 Suppresses Cell Apoptosis by Targeting PDCD4. Int J Mol Sci. 2019 Jun 1;20(11).

3. Li T, Morgan MJ, Choksi S, et al. MicroRNAs modulate the noncanonical transcription factor NF-kappaB pathway by regulating expression of the kinase IKKalpha during macrophage differentiation. Nat Immunol. 2010 Sep;11(9):799-805.

4. Bi J, Zeng X, Zhao L, et al. miR-181a Induces Macrophage Polarized to M2 Phenotype and Promotes M2 Macrophage-mediated Tumor Cell Metastasis by Targeting KLF6 and C/EBPalpha. Mol Ther Nucleic Acids. 2016 Sep 27;5(9):e368.

5. Xie W, Li M, Xu N, et al. MiR-181a regulates inflammation responses in monocytes and macrophages. PLoS One. 2013;8(3):e58639.

6. Jiang K, Guo S, Zhang T, et al. Downregulation of TLR4 by miR-181a Provides Negative Feedback Regulation to Lipopolysaccharide-Induced Inflammation. Front Pharmacol. 2018;9:142.

7. Wu Z, Chen J, Zhao W, et al. Inhibition of miR-181a attenuates sepsis-induced inflammation and apoptosis by activating Nrf2 and inhibiting NF-kappaB pathways via targeting SIRT1. Kaohsiung J Med Sci. 2021 Mar;37(3):200-207.

8. Marisetty A, Wei J, Kong LY, et al. MiR-181 Family Modulates Osteopontin in Glioblastoma Multiforme. Cancers (Basel). 2020 Dec 17;12(12).

9. Chistiakov DA, Orekhov AN, Bobryshev YV. Chemokines and Relevant microRNAs in the Atherogenic Process. Mini Rev Med Chem. 2018;18(7):597-608.

10. Fayyad-Kazan H, Hamade E, Rouas R, et al. Downregulation of microRNA-24 and -181 parallels the upregulation of IFN-gamma secreted by activated human CD4 lymphocytes. Hum Immunol. 2014 Jul;75(7):677-85.

11. Ghorbani S, Talebi F, Chan WF, et al. MicroRNA-181 Variants Regulate T Cell Phenotype in the Context of Autoimmune Neuroinflammation. Front Immunol. 2017;8:758.

12. Zhu LM, Yang M. The suppression of miR-181 inhibits inflammatory responses of osteoarthritis through NF-kappaB signaling pathway. Eur Rev Med Pharmacol Sci. 2019 Jul;23(13):5567-5574.

13. Su R, Lin HS, Zhang XH, et al. MiR-181 family: regulators of myeloid differentiation and acute myeloid leukemia as well as potential therapeutic targets. Oncogene. 2015 Jun;34(25):3226-39.

14. Mei F, Wang J, Chen Z, et al. Potentially Important MicroRNAs in Form-Deprivation Myopia Revealed by Bioinformatics Analysis of MicroRNA Profiling. Ophthalmic Res. 2017;57(3):186-193.
